# Lipotoxic stress alters the membrane lipid profile of extracellular vesicles released by Huh-7 hepatocarcinoma cells

**DOI:** 10.1038/s41598-021-84268-9

**Published:** 2021-02-25

**Authors:** Sandra Buratta, Y. Shimanaka, E. Costanzi, S. Ni, L. Urbanelli, N. Kono, F. Morena, K. Sagini, S. Giovagnoli, R. Romani, M. Gargaro, H. Arai, C. Emiliani

**Affiliations:** 1 Department of Chemistry, Biology and Biotechnology, University of Perugia, Perugia, Italy; 2 Graduate School of Pharmaceutical Sciences, University of Tokyo, Tokyo, Japan; 3 Department of Molecular Cell Biology, Institute for Cancer Research, Oslo University Hospital, Oslo, Norway; 4 Department of Pharmaceutical Sciences, University of Perugia, Perugia, Italy; 5 Department of Experimental Medicine, University of Perugia, Perugia, Italy; 6 AMED-CREST, Japan Agency for Medical Research and Development, Tokyo, Japan

**Keywords:** Cell signalling, Lipids

## Abstract

Extracellular vesicles (EVs) are well-known mediators in intercellular communication playing pivotal roles in promoting liver inflammation and fibrosis, events associated to hepatic lipotoxicity caused by saturated free fatty acid overloading. However, despite the importance of lipids in EV membrane architecture which, in turn, affects EV biophysical and biological properties, little is known about the lipid asset of EVs released under these conditions. Here, we analyzed phospholipid profile alterations of EVs released by hepatocarcinoma Huh-7 cells under increased membrane lipid saturation induced by supplementation with saturated fatty acid palmitate or Δ9 desaturase inhibition, using oleate, a nontoxic monounsaturated fatty acid, as control. As an increase of membrane lipid saturation induces endoplasmic reticulum (ER) stress, we also analyzed phospholipid rearrangements in EVs released by Huh-7 cells treated with thapsigargin, a conventional ER stress inducer. Results demonstrate that lipotoxic and/or ER stress conditions induced rearrangements not only into cell membrane phospholipids but also into the released EVs. Thus, cell membrane saturation level and/or ER stress are crucial to determine which lipids are discarded via EVs and EV lipid cargos might be useful to discriminate hepatic lipid overloading and ER stress.

## Introduction

Extracellular vesicles (EVs) are nanometric membrane-surrounded particles representing a cellular route to pack and transfer specific molecular cargo from donor to recipient cells, in both physiological and pathological status^1,2^. Cells release two main types of particles, exosomes (Ex), originating by late endosomes full of small intraluminal vesicles (ILVs) named multivesicular bodies (MVBs), and microvesicles (MVs) originating by the outward budding of plasma membranes. However, Ex and MVs share partially overlapping biophysical characteristics (i.e. size and density) and when considering experimentally obtained isolates, current protocols cannot discriminate between these two and other vesicle subtypes^3^.

EV formation, secretion, and uptake by target cells occur through highly regulated mechanisms that require the package of specific molecules, including certain lipid classes^4,5^. Different lipid-related pathways are involved in the EV biogenesis^6^. A functional role is played by phosphatidic acid (PtdA), a small negatively charged headgroup phospholipid that favors the invagination of endosomal membranes forming ILVs, and cholesterol, that regulates MVBs transfer towards the plasma membranes^5^. Another cone-shaped lipid that favors the invagination of the endosomal membrane is ceramide (Cer)^7^.

As common features, independently of their cell origin, in comparison with their parental cells, EVs are enriched in cholesterol, glycosphingolipids, ether-linked lipids, and lysosophospholipids^4,5,8–11^. In particular, the presence of ether lipids in EVs might have important implications for both fusion efficiency and stability in the extracellular space^12^. Another characteristic of EVs is the enrichment in membrane phospholipids containing saturated fatty acids compared to originating cells^8,9,13^. EV phospholipids also represent sources of lipid mediators partially synthesized by enzymes packaged within vesicles^14^. Conveying bioactive lipids via EVs represents a mean to protect these mediators from degradation and exchange these molecules between different cell types, thus playing a pivotal role in their transcellular biosynthesis^14^.

A growing number of reports show that EVs play important roles in liver pathophysiology mediating the intercellular communication among different liver cell populations as well as between liver and other tissues via circulation^15^. EVs have been particularly investigated in nonalcoholic fatty liver disease (NAFLD), a chronic liver disease characterized by hepatic steatosis without excess alcohol consumption, and in non-alcoholic steatohepatitis (NASH), a disease characterized by hepatocellular steatosis accompanied by inflammation and fibrosis as a result of prolonged NAFLD^15,16^. An increased EV release has been demonstrated either in in vitro models of lipotoxicity and in in vivo models of NASH^17–20^. The treatment of human and mouse hepatocytes with palmitic acid (PA, C16:0), detected at high concentrations in NAFLD livers, induces the release of Cer-enriched EVs, which exhibit proinflammatory effects by the activation of macrophage chemiotaxis^16–20^. Then, a set of miRNAs carried by hepatic lipotoxic EVs seems to be responsible for the activation of hepatic stellate cells (HSCs), an event responsible for liver fibrosis occurring in NAFLD^21^. All these studies suggest a mechanistic link between hepatic lipotoxicity and macrophage and HSCs recruitment to the steatotic liver during NASH development.

To date, no studies have evaluated the global lipid composition of EVs released by hepatocytes under lipotoxicity, despite the importance of lipid cargo of EVs released under these conditions. Biological effects exerted by hepatic lipotoxic EVs could be also mediated by specific lipid species present on their membranes or by products of phospholipid precursors. Further, EV lipid profiles might mirror rearrangements of cellular membrane phospholipids occurring during hepatic lipid overloading. Thus, EVs possessing specific lipid composition might be useful as markers for monitoring hepatic lipotoxicity^19^.

Here, we evaluated lipid profiles of EVs released by human hepatoma Huh-7 cells under conditions that increase levels of saturated fatty acids in membrane phospholipids. The increase of membrane lipid saturation was promoted by treating Huh-7 cells with PA or with an inhibitor of stearoyl-CoA desaturase-1 (SCD1i), an enzyme that catalyzes the Δ9-desaturation of PA and stearic acid (18:0) forming the corresponding monounsaturated fatty acids (i.e. palmitoleic acid (16:1n-7) and oleic acid (OA, 18:1n-9)). These are well accepted in vitro models for evaluating molecular mechanisms underlying hepatic lipotoxicity^17–19,22,23^. Considering that an increase of membrane lipid saturation induces ER stress^22,23^, we also analyzed lipid composition of EVs released by Huh-7 cells treated with Thapsigargin (Tg), a conventional ER stress inducer, as well as that of EVs released by Huh-7 cells treated with OA, a non-toxic monounsaturated fatty acid.

Our findings provide a complete picture of EV phospholipid profile alterations occurring during membrane lipid saturation and/or ER stress conditions. In particular, the three ER stressors (PA, SCD1i, Tg) induced rearrangements in both cell and EV membrane phospholipids. EVs released upon different treatments could be discriminated by their lipid profiles (i.e. PA-EVs from SCD1i-EVs and Tg-EVs). Finally, we show that EVs released by treated cells presented different internalization efficiency by target cells compared to EVs from control cells.

## Results

### Characterization of EVs released by Huh-7 cells under membrane lipid saturation and/or ER stress conditions

**Figure 1:**
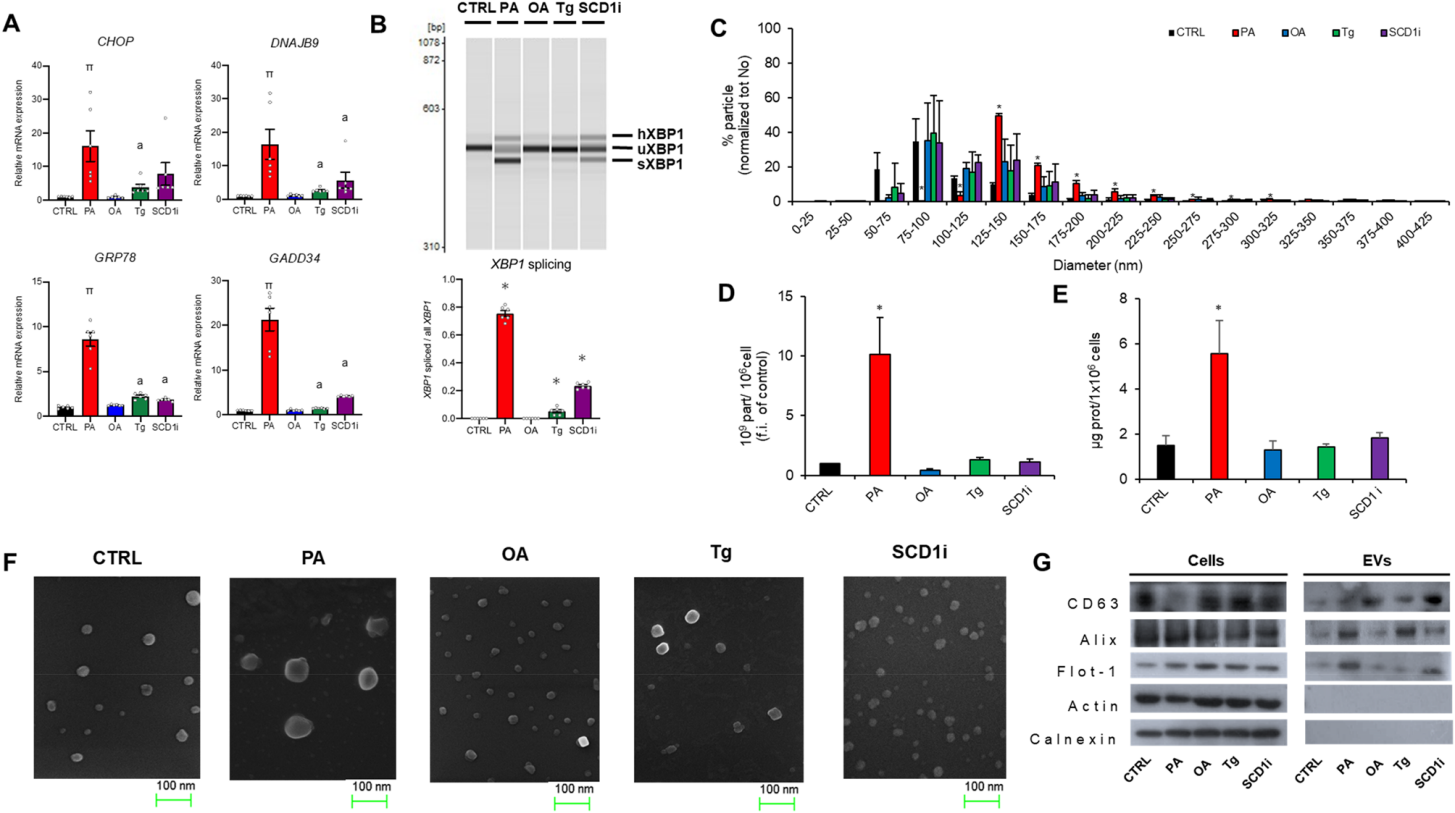
Characterization of EVs released from Huh-7 cells under membrane lipid saturation and/or ER stress conditions. EVs were isolated from culture media of Huh-7 cells treated for 16 h with 400 µM of fatty acids (PA or OA), or 2.5 nM Tg, or 5 µM SCD1i or vehicle (CTRL). (**A**) Expression of UPR-target genes. At the end of incubation, total RNA was extracted and CHOP and DNAJB9, GADD34 and GRP78 mRNAs were quantified by qRT-PCR. The expression level of each gene was normalized to the GAPDH gene and is represented as fold induction over CTRL. Data are reported as mean ± SD (n = 6); *p* < 0.05 was considered statistically significant by one-way ANOVA and Tukey’s post-hoc analysis; columns with different letters are significantly different (i.e. a vs PA and π vs all). (**B**) XBP1 splicing measured by RT-PCR and electrophoresis in Huh-7 cell treated with each reagent. (Top) The representative image of electrophoresis. The positions of spliced form (XBP1S), unspliced form (XBP1U), and heteroduplex of these forms (XBP1U + S) are indicated. (Bottom) XBP1 splicing was quantified as the value of the XBP1S over the total XBP1 detected. Data are reported as mean ± SD (n = 6); * p < 0.05 was considered statistically significant to all by one-way ANOVA and Tukey’s post-hoc analysis. C) Size distribution of released EVs by NTA shown the percentage of particles normalized by total number of particles for each condition. Data are the mean ± SD of three preparations. (**D**) Quantification of the released EVs by NTA. Data are expressed as mean ± SD of three preparations (Student’s multi-t-test * p < 0.05, treated vs CTRL). (**E**) Recovered EVs quantified as μg proteins/10^6^ cells. Values are the mean ± SD of five preparations (Student’s multi-t-test * p < 0.05, treated vs CTRL). (**F**) Representative images of EVs by SEM; (**G**) Western blotting for EV markers. Cell extracts (15 μg) and EVs (2 μg) were separated by SDS-PAGE, electrotransferred and probed with the indicated positive and negative markers of EVs. Western blots are representative of two independent experiments.

To characterize EVs released under membrane lipid saturation and/or ER stress conditions, Huh-7 cells were treated for 16 h with PA (400 μM) or with a SCD1 inhibitor (CAY 10,566, 5 μM)^24^ to increase the saturation level of membrane phospholipids, or Tg (2.5 nM), to induce classical ER stress^25^. Huh-7 cells incubated with vehicle (CTRL) or with OA (400 μM)^19^ were used as controls. The concentration of fatty acids, Tg and SCD1i and the period of treatment were chosen on the basis of previous studies on Huh-7 cells^18,19^. In agreement with literature data, these experimental conditions did not affect significantly cell viability (data not shown). PA, SCD1i and Tg induced ER stress in Huh-7 cells as demonstrated by the increased expression of four representative unfolded protein response (UPR) target genes, C/EBP Homologous Protein (CHOP), DnaJ Heat Shock Protein Family Member B9 (DNAJB9), growth arrest and DNA damage inducible protein 34 (GADD34) and glucose-regulated protein 78 (GRP78) (Fig. 1A). We also examined activation of the UPR sensor protein inositol-requiring 1 (IRE1). PA, SCD1i and Tg treatment activated IRE1 as evidenced by splicing of Xbox-binding protein 1 (XBP1) mRNA (Fig. 1B). PA treatment caused the most severe ER stress among the three stressors (PA, SCD1i and Tg).

Then, EVs were isolated from culture media of cells by differential centrifugation^26^, which includes a final step of high-speed ultracentrifugation step that permit to obtain vesicle preparations enriched in small sized EVs^26,27^. In fact, Nanoparticle Tracking Analysis (NTA) showed that the size distribution of CTRL- EVs was between 50 and 200 nm (Fig. 1C) with a mode size of 84 ± 8.2 nm, consistent with exosome and/or small membrane-derived vesicles. Interestingly, only PA treatment increased the mode size (143 ± 8.4 nm) of released EVs, while no changes were observed in EV populations from OA, Tg or SCD1i-treated cells, compared to CTRL-EVs (Fig. 1C). As the percentage of particles with size ≥ 150 nm was less than 10% in all experimental groups, large EVs or aggregation products of small EVs were negligible in our EV preparations.

**Figure 2:**
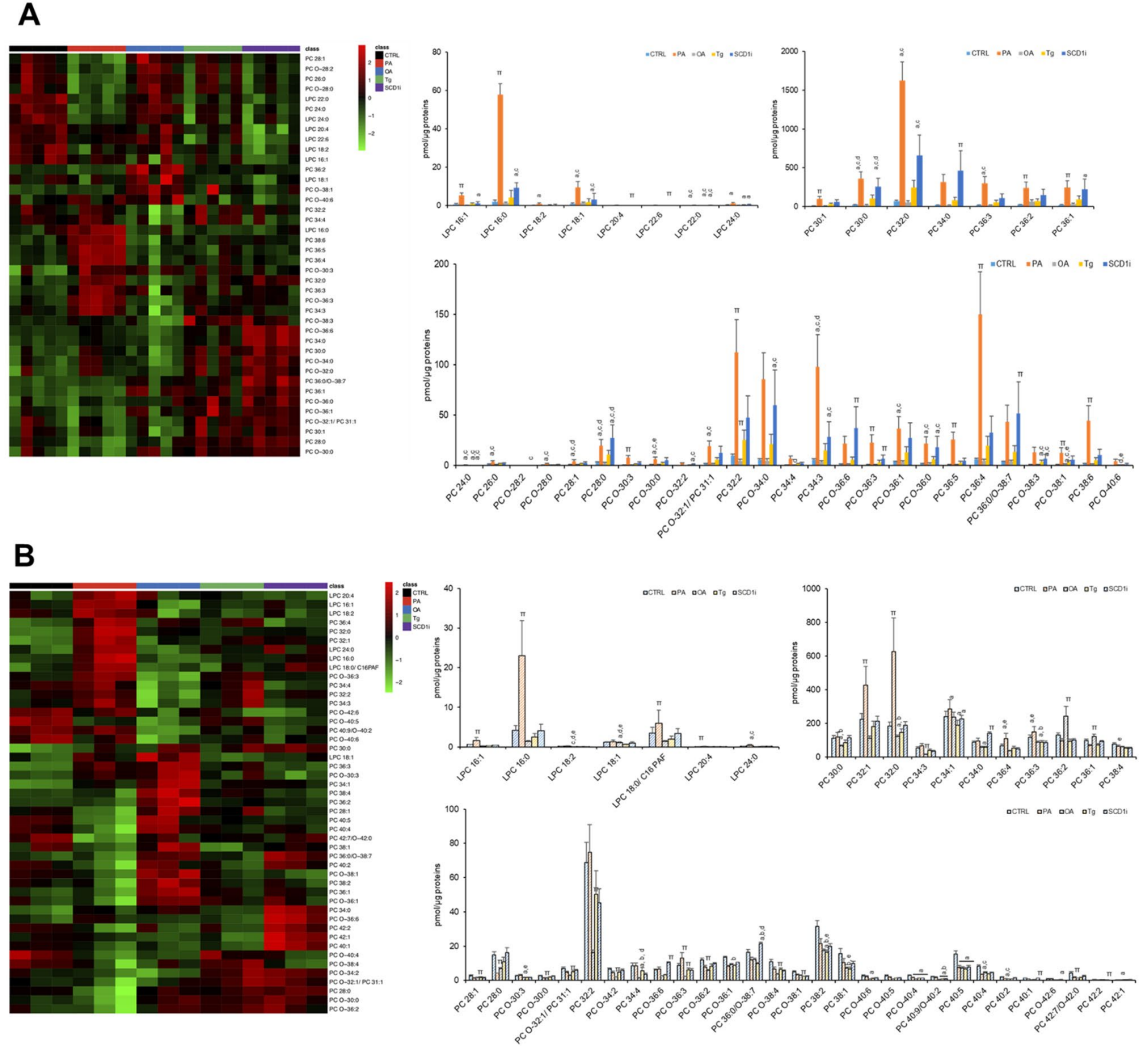
Effects of treatment with fatty acids (PA or OA), SCD1 inhibitor or Tg on the levels of PCs and LPCs in Huh-7 cells and their released EVs. Lipid extracts from EVs (**A**) and from their releasing cells (**B**) were analyzed by LC/MS–MS. Heatmap of clustering results showing molecular species significantly changed among all the PCs and LPCs detected. Each rectangle represents a lipid colored by its normalized intensity scale from green (decreased level) to red (increased level). The Euclidean metric for distance measurement and the Ward.D algorithm for hierarchical clustering were used. Bar graphs show changes in the abundance of individual lipid species. Data are reported as mean ± SD (n = 3, cells; n = 5, EVs); *p* < 0.05 was considered statistically significant by one-way ANOVA and Tukey’s post-hoc analysis; columns with different letters are significantly different (i.e. a vs CTRL, b vs PA, c vs OA, d vs Tg, e vs SCD1i and π vs all).

The amount of released EVs was measured by NTA and normalized with respect to cell number. As shown in Fig. 1D, only PA treatment induced an increased EV release (~ tenfold). Consistently, a greater protein amount was recovered in the pellet obtained from ultracentrifugation of PA-treated cell culture media (Fig. 1E). Scanning Electron Microscopy (SEM) analysis showed a rounded morphology and a diameter of the released EVs generally below 100 nm, characteristic typical of vesicle populations enriched in Ex of endosomal origin (Fig. 1F). Immunoblotting analysis demonstrated the presence of EV positive markers (CD63, Alix and Flot-1)^27^ (Fig. 1G). The absence of calnexin indicated that these EV preparations did not contain a detectable quantity of cell debris. Actin, investigated as loading control in cells, was not detected in our EV samples. An enrichment of CD63 and depletion of calnexin in EVs were also demonstrated by loading a similar amount of proteins from cell lysates and EVs on the same Western blotting (Fig. 1S). Finally, apo B was used to evaluate the lipoprotein contamination in our EV preparations; as shown in Fig. 2S, apo B was absent in EV released by control and treated cells.

These results demonstrated that our preparations contain mostly small EVs, that we generically named EVs in the rest of the article. Furthermore, among treatments triggering ER stress, only PA increased both the number and size of released EVs.

### Lipid analysis of EVs released by Huh-7 cells under membrane lipid saturation and/or ER stress conditions

Lipid analysis of EVs and their originating cells was carried out by liquid chromatography coupled with tandem mass spectrometry (LC–MS/MS). A detailed evaluation of changes in molecular species within individual phospholipid classes was reported in both EVs and cells. For each phospholipid class, heatmaps show lipid species whose levels were statistically different among five experimental groups, whereas bar graphs illustrate differences in the abundance of individual lipid species.

The analysis of glycerophospholipids in EVs with choline as polar head identified 32 phosphatidylcholines (PC) and 8 lyso-phosphatidylcholines (LPC) whose levels were significantly changed among five experimental groups (Fig. 2A). Major changes were observed in PA-EVs and in SCD1i-EVs that were characterized by increased levels of 22 and 14 PC molecular species, respectively, compared to other experimental groups (Fig. 2A). Once more, major differences were found for LPC in PA-EVs (LPC 16:0, 16:1; 22:0 and 24:0 increased), SCD1i-EVs (LPC 16:0, 22:0; 24:0 increased; LPC 20:4 decreased), but also in OA-EVs (LPC 18:1 increased; LPC 18:2 decreased) (Fig. 2A). In cells, levels of 40 PCs and 7 LPCs were statistically different among five experimental groups (Fig. 2B). Rearrangements in PC acyl chains were also observed in PA (7 PC species increased and 8 PC species decreased) and in SCD1i-treated cells (5 PC species increased and 5 PC species decreased) (Fig. 2B). In OA-treated cells, levels of two PC species (36:1 and 36:2) increased and levels of 21 PC species decreased, compared to other experimental groups (Fig. 2B). For LPC, mostly fatty acid rearrangements were observed in PA-treated cells (LPC 16:0, 16:1, 18:0/C16 PAF, 18:2, 20:4; 24:0 increased).

**Figure 3:**
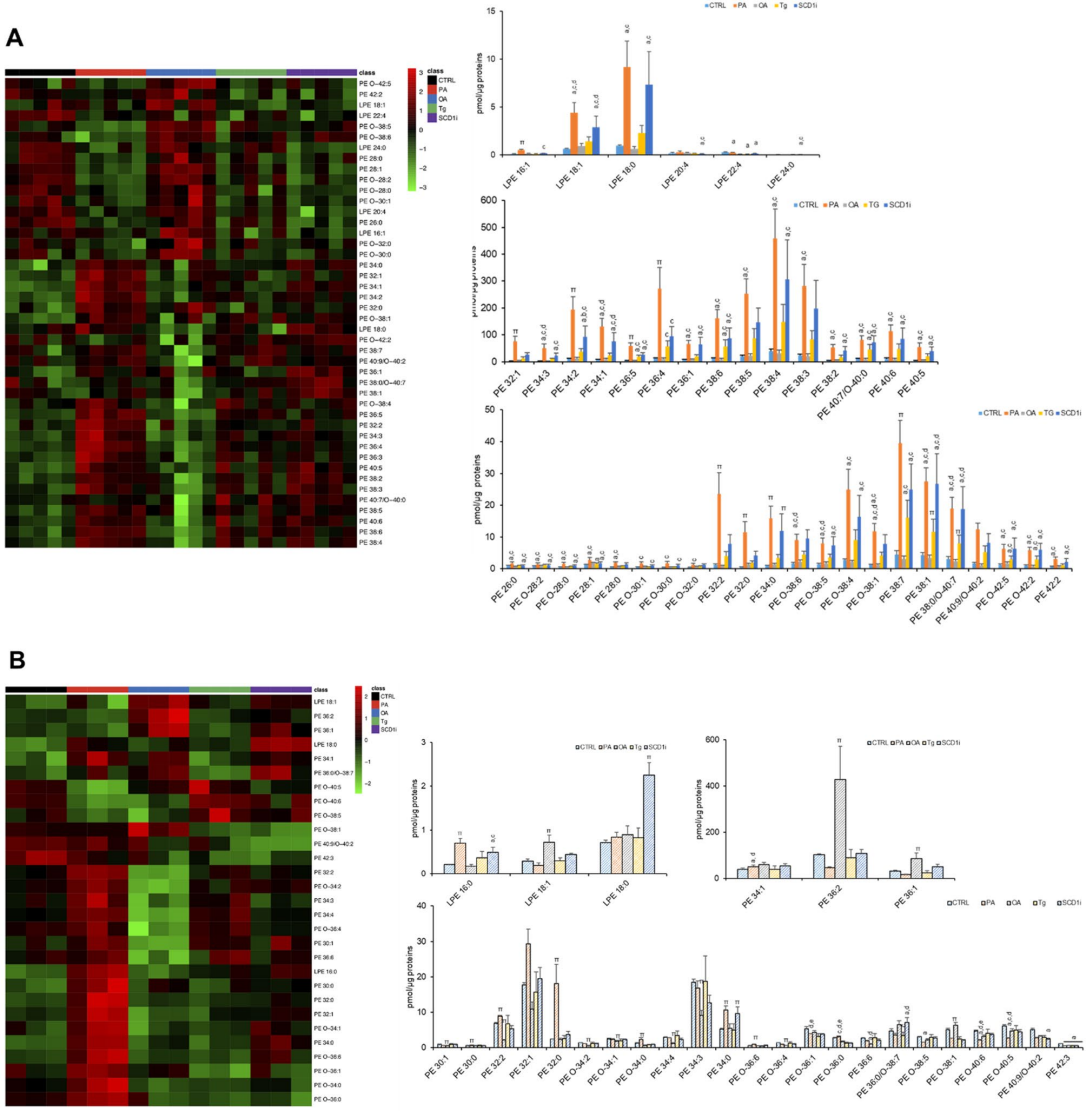
Effects of treatment with fatty acids (PA or OA), SCD1 inhibitor or Tg on the levels of PEs and LPEs in Huh-7 cells and their released EVs. Lipid extracts from EVs (**A**) and from their releasing cells (**B**) were analyzed by LC/MS–MS. Heatmap of clustering results showing molecular species significantly changed among all PEs and LPEs detected. Each rectangle represents a lipid colored by its normalized intensity scale from green (decreased level) to red (increased level). The Euclidean metric for distance measurement and the Ward.D algorithm for hierarchical clustering were used. Bar graphs show changes in the abundance of individual lipid species. Data are reported as mean ± SD (n = 3, cells; n = 5, EVs); *p* < 0.05 was considered statistically significant by one-way ANOVA and Tukey’s post-hoc analysis; columns with different letters are significantly different (i.e. a vs CTRL, b vs PA, c vs OA, d vs Tg, e vs SCD1i and π vs all).

As for ethanolamine-containing glycerophospholipids in EVs, we observed that 38 phosphatidylethanolamines (PE) were statistically different among the five EV groups (Fig. 3A) and, interestingly, they resulted increased in PA-EVs compared to CTRL-EVs and OA-EVs. Likewise, 26 PE species and 10 PE species were increased in SCD1i-EVs and in Tg-EVs, respectively. Rearrangements in 6 species of lyso-phosphatidylethanolamine (LPE) were observed in PA- EVs (LPE 16:1; 18:0; 18:1 increased and LPE 22:4 decreased) and in SCD1i- EVs (LPE 18:0; 18:1; 24:0 increased and LPE 20:4, 22:4 decreased) (Fig. 3A). In cells, major changes were observed in PA (9 PE species increased and 4 PE species decreased), SCD1i (3 PE species increased and 3 PE species decreased) and OA treated cells (3 PE species increased and 9 PE species decreased) (Fig. 3B). SCD1i treatment induced an increased level of LPE 16:0 and 18:0, whereas PA treatment a higher level of LPE 16:0. An enrichment of LPE 18:1 was observed in OA-treated cells (Fig. 3B).

**Figure 4:**
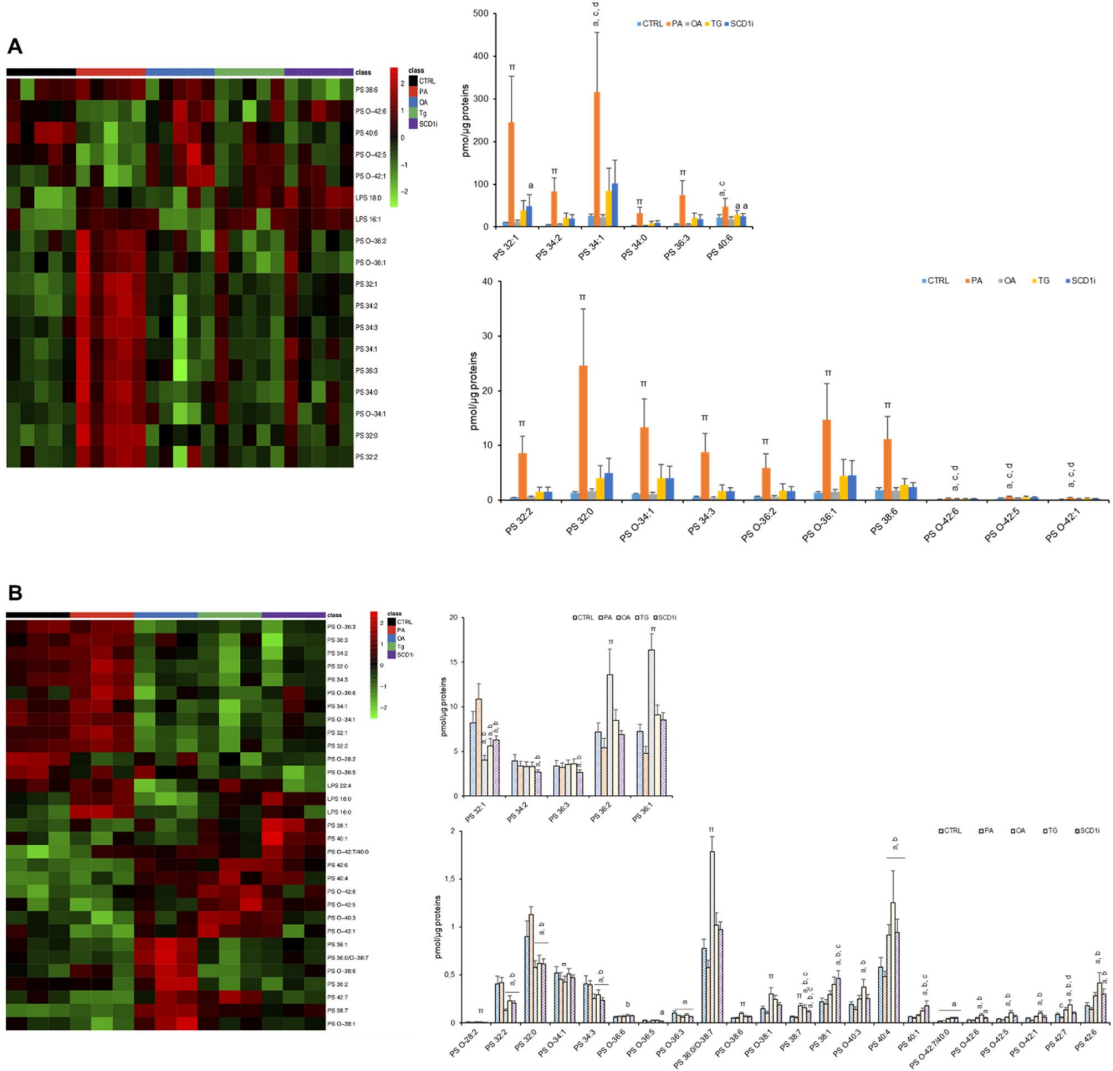
Effects of treatment with fatty acids (PA or OA), SCD1 inhibitor or Tg on the levels of PSs and LPSs in Huh-7 cells and their released EVs. Lipid extracts from EVs (**A**) and from their releasing cells (**B**) were analyzed by LC/MS–MS. Heatmap of clustering results showing molecular species significantly changed among all PSs detected. Each rectangle represents a lipid colored by its normalized intensity scale from green (decreased level) to red (increased level). The Euclidean metric for distance measurement and the Ward.D algorithm for hierarchical clustering were used. Bar graphs show changes in the abundance of individual lipid species. Data are reported as mean ± SD (n = 3, cells; n = 5, EVs); *p* < 0.05 was considered statistically significant by one-way ANOVA and Tukey’s post-hoc analysis; columns with different letters are significantly different (i.e. a vs CTRL, b vs PA, c vs OA, d vs Tg, e vs SCD1i and π vs all).

Figure 4 shows a detailed analysis of phosphatidylserines (PS) and lyso-phosphatidylserines (LPS). In EVs, 16 PS species and 2 LPS species were significantly different among five experimental groups (Fig. 4A). Specifically, PA-EVs were characterized by higher levels of all the 16 PS species whereas SCD1i-EVs presented higher levels of 3 PS species (32:0; 32:1 and 40:0), compared to CTRL-EVs (Fig. 4A). PA-EVs and SCD1i-EVs presented also increased levels of LPS 16:0 and 18:0. In cells, significant changes in the level of 28 PS species and 3 LPS species were detected (Fig. 4B). Regarding PS, major changes were observed in cells treated with OA, Tg and SCD1i compared to the CTRL (Fig. 4B). The level of LPS 16:0 and 18:0 was increased in PA and SCD1i-treated cells, whereas LPS 22:4 was increased in Tg-treated cells.

None of the four treatments was able to induce acyl chain rearrangements in the less abundant phospholipid classes, i.e. phosphatidylinositol (PI), phosphatidylglycerol (PG) and phosphatidic acid (PtdA) (data not shown).

**Figure 5:**
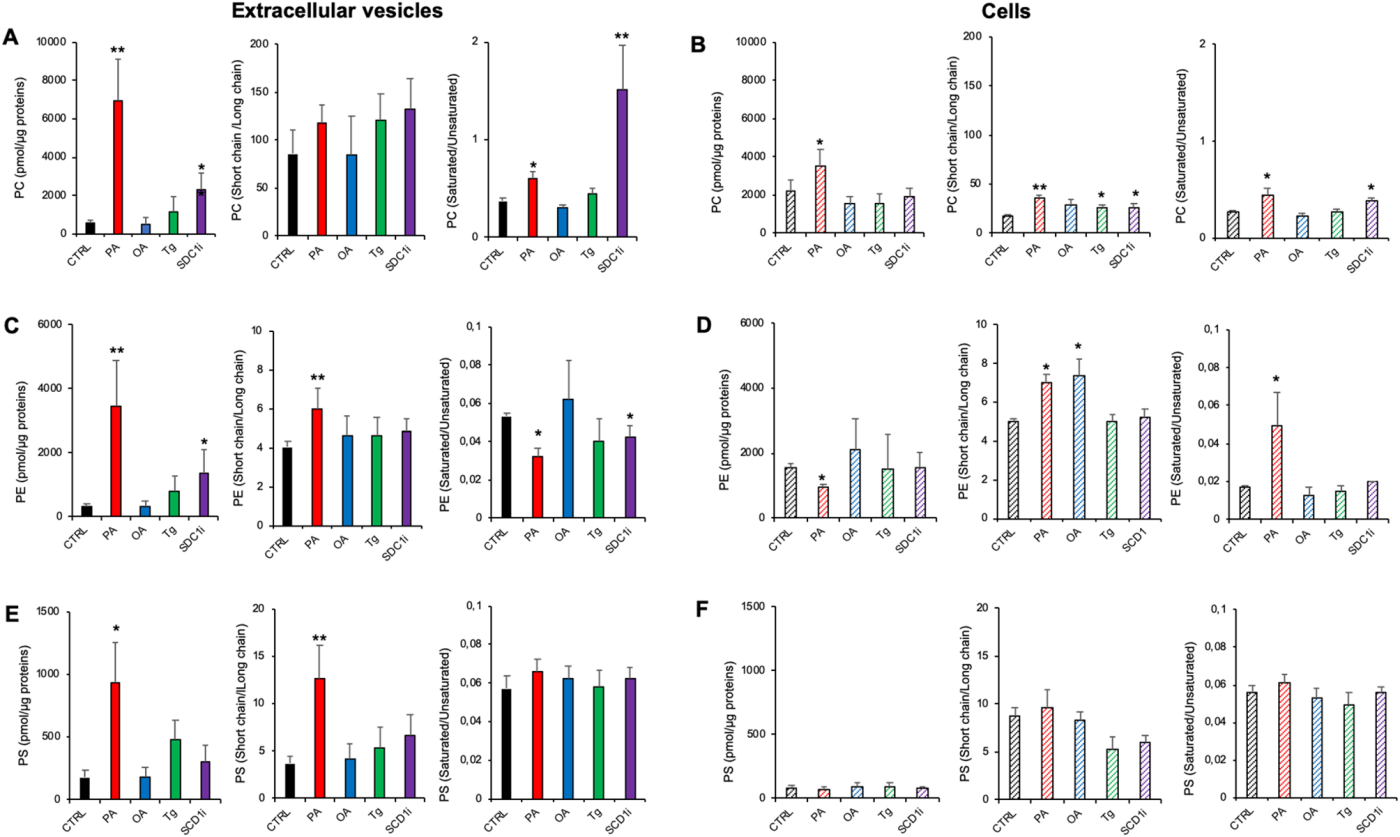
Characterization of phospholipid classes and fatty acid ratios of Huh-7 cells treated with fatty acids (PA or OA), SCD1 inhibitor or Tg, and of their released EVs. Panels A, C and E show data relative to EVs. Panels B, D and F show data relative to cells. (**A**, **B**) PC content (sum of all detected species) expressed as pmol/μg of proteins; ratio of PCs with acyl chains whose sum was < 36 carbons (short chain) to PCs acyl chains whose sum was > 38 carbons (long chains); ratio of saturated to unsaturated PC species. (**C**, **D**) PE content (sum of all detected species) expressed as pmol of lipid species/μg of proteins; ratio of PEs with acyl chains whose sum was < 36 carbons (short chain) to PEs with acyl chains whose sum was > 38 carbons (long chains); ratio of saturated to unsaturated PE species. (**E**, **F**) PS content (sum of all detected species) expressed as pmol of lipid species/μg of proteins; ratio of PSs with acyl chains whose sum was < 36 carbons (short chain) to PSs with acyl chains whose sum was > 38 carbons (long chains); ratio of saturated to unsaturated PS species. Mean values ± SD (n = 5, EVs; n = 3, cells) are shown (**p < 0.01, * p < 0.05, treated vs CTRL; ANOVA followed with a Dunnett’s multiple comparisons test).

To evaluate the impact of acyl chain rearrangements on the phospholipid properties, we calculated the length and saturation degree of acyl chains in PC, PE and PS, for both EVs and cells. Figure 5A,B show the total PC content normalized for protein content, the ratio of short acyl chains (sum of carbons < 36) versus long acyl chains (sum of carbons > 38) containing PC species, and the ratio of saturated versus unsaturated PC species. A greater content of PC and an enrichment in PC saturated species were observed in PA-EVs and SCD1i-EVs, compared to CTRL-EVs (Fig. 5A). In cells, we observed that: a) total PC content was increased in PA-treated cells; b) levels of shorter acyl chain PC species were increased in PA, Tg and SCD1i-treated cells; c) levels of saturated PCs were increased in PA and SCD1i-treated cells (Fig. 5B). A greater content of total PE accompanied by a reduced level of PE containing saturated species was observed in PA-EVs and SCD1i-EVs, compared to CTR-EVs. A greater level of PE containing short acyl chains characterized PA-EVs (Fig. 5C). In cells, PA treatment reduced PE content and increased the level of saturated PE. PEs containing short acyl chains were increased in PA and OA-treated cells as well (Fig. 5D). Figure 5E,F show the total asset of PS in both EVs and cells, respectively. A higher content of PS and higher ratio of short versus long fatty acids characterized PA-EVs (Fig. 5E). In cells, no treatments induced relevant rearrangements in the length and saturation degree of PS acyl chains (Fig. 5F).

**Figure 6:**
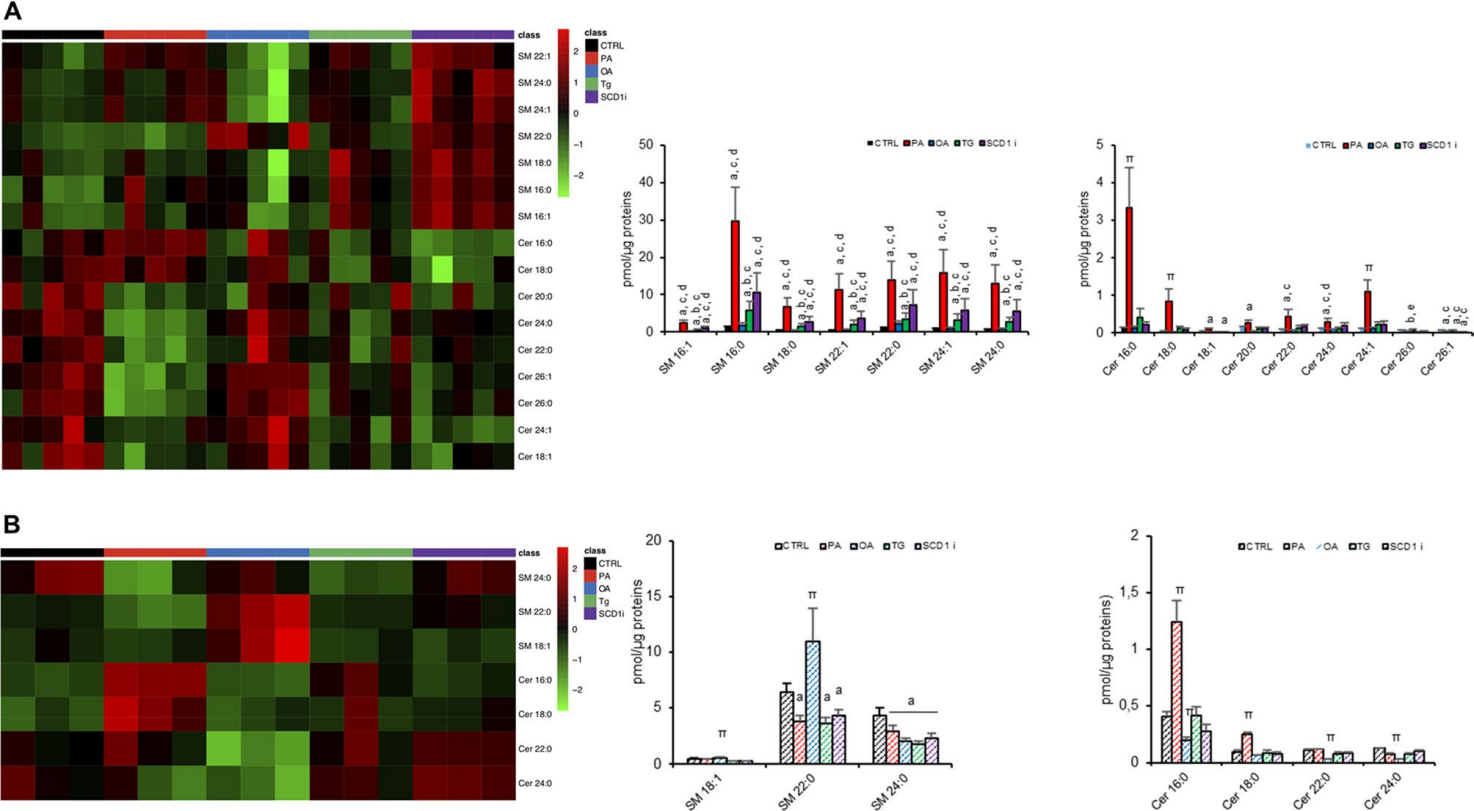
Effects of treatment with fatty acids (PA or OA), SCD1 inhibitor or Tg on the levels of SM and Cer in Huh-7 cells and their released EVs. Heatmap of clustering results showing the molecular species of SM and Cer significantly changed among all lipid species detected. Each rectangle represents a lipid colored by its normalized intensity scale from green (decreased level) to red (increased level). The Euclidean metric for distance measurement and the Ward.D algorithm for hierarchical clustering were used. Bar graphs show changes in the abundance of individual lipid species. Data are reported as mean ± SD (n = 3, cells; n = 5, EVs); *p* < 0.05 was considered statistically significant by one-way ANOVA and Tukey’s post-hoc analysis; columns with different letters are significantly different (i.e. a vs CTRL, b vs PA, c vs OA, d vs Tg, e vs SCD1i and π vs all).

Finally, we reported detailed changes within sphingomyelins (SM) and Cer(s) in EVs and their originating cells (Fig. 6). In PA-EVs, Tg-EVs and SCD1i-EVs, 7 SMs were increased, compared to CTRL-EVs and OA-EVs (Fig. 6A). In PA-EVs we observed also increased levels of 7 Cer(s). In SCD1i-EVs, levels of Cer 18:1 and Cer 26:1 were decreased (Fig. 6A). In cells, 2 SMs (18:1, 22:0) were increased in OA-treated cells, while SM 22:0 and SM 24:0 were decreased in PA, Tg and SCD1i-treated cells compared to CTRL (Fig. 6B). PA treatment increased Cer 16:0 and 18:0 levels, while in OA treatment decreased Cer 16:0, 22:0 and 24:0 levels (Fig. 6B).

**Figure 7:**
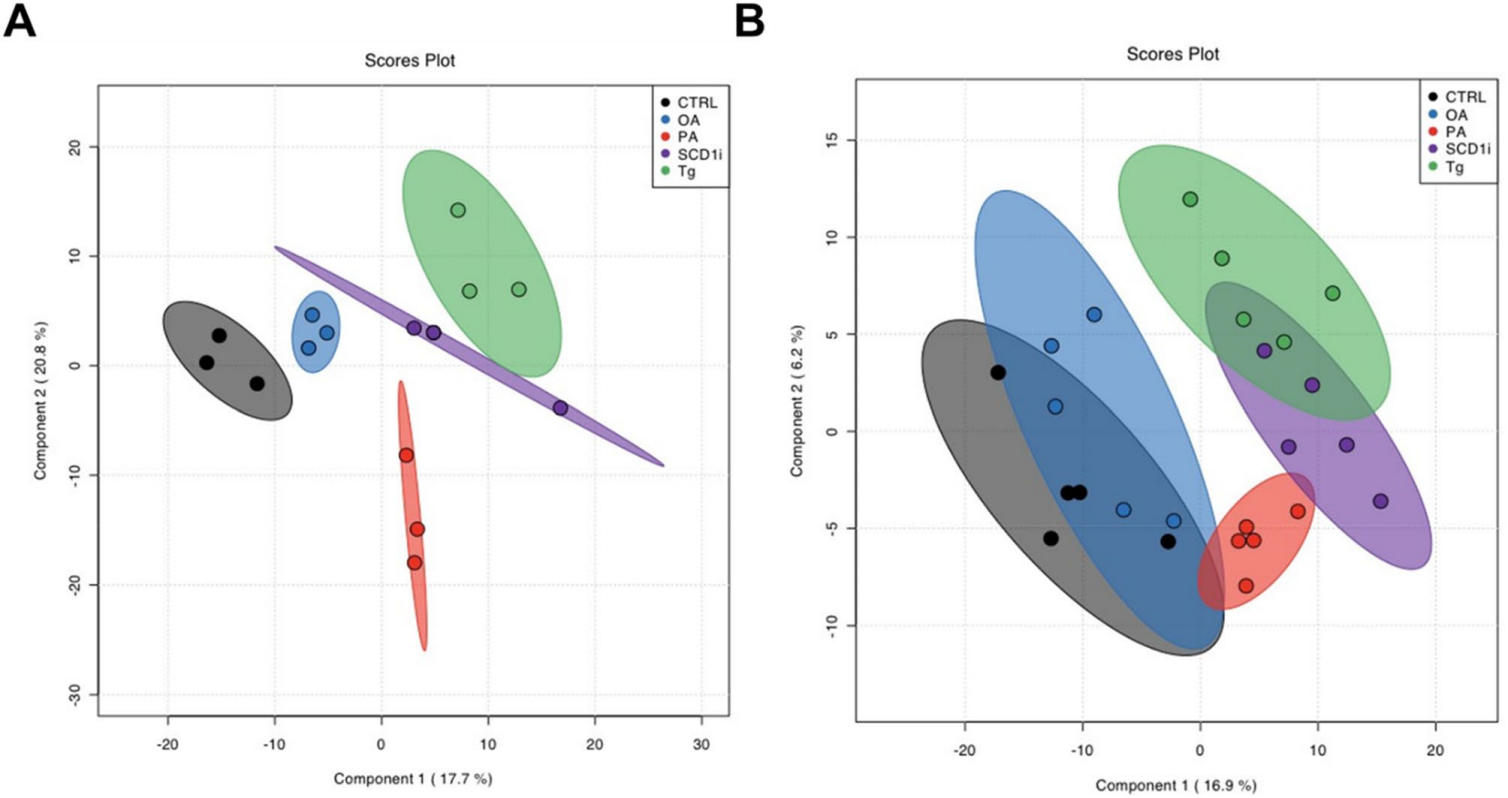
Multivariate PLS-DA of the lipidomic dataset. PLS-DA analysis of all lipid species identified from control and treated cells (**A**) and their released EVs (**B**). Treated groups versus control group. PLS-DA was applied to the cleaned and log-transformed dataset. CI 95% ellipses are shown for the five different groups.

Partial Least Squares Discriminant Analysis (PLS-DA), a supervised multi-dimensional statistical model analysis, was applied to analyze the five experimental groups based on the whole lipid data, both for cells and EVs. Regarding cells, PLS-DA score plot indicated that the five experimental groups can be separated according to the treatment, based on their lipid profiles (Fig. 7A). Specifically, component 1 separated PA, Tg and SCD1i-treated cells from CTRL and OA-treated cells, and component 2 separated PA-treated cells from the other experimental groups (Fig. 7A). The goodness-of-fit (R2) of 0.92 indicated that PLS-DA was a reliable model to detect differences among the five sample groups, and the goodness-of-prediction (Q2) of 0.58, together with a 2000-time permutation test (*p* = 0.0055), confirmed an adequate robustness.

On the other hand, the PLS-DA analysis of EVs displayed that the five lipidomic datasets clustered into three main groups. PA-EVs clustered in a separated group, indicating that PA-EVs exhibited a very distinct lipid composition (Fig. 7B). Tg-EVs and SCD1i-EVs were not clearly separated from each other but were well separated from the CTRL-EVs and OA-EVs (Fig. 7B). These results clearly show that ER stress induced a common response in terms of EV lipid composition (Tg-EVs and SCD1i-EVs), but PA treatment shows additional peculiar features. Even in this case, according to the goodness-of-fit (R2) of 0.90, PLS-DA can be considered an adequate model to detect differences among the five sample groups. Nevertheless, the goodness-of-prediction (Q2) of 0.31 revealed a lower predictive ability, even with a positive 2000-time permutation test (*p* = 0.0085).

Data were also analyzed using Random Forests (RF), a machine learning method widely used to identify variables which best classify the data into different groups. The RF analysis demonstrated that the five groups of cells were well classified in agreement with the PLS-DA analysis (Fig. 3S, Table 1S). In particular, CTRL, OA, and PA-treated cells were clearly classified while Tg and SCD1i-treated cells are mispredicted in 33% of cases (Table 1S). In EVs, RF analysis showed that the CTRL-EVs and OA-EVs had similar lipid profiles whereas PA-EVs had a characteristic and distinct lipid composition. SCD1i-EVs and Tg-EVs were not clearly classified with a classification error of 0.4 and 0.8, respectively (Fig. 4S, Table 2S).

### Uptake of Huh-7-derived EVs by THP-1 monocytes

**Figure 8:**
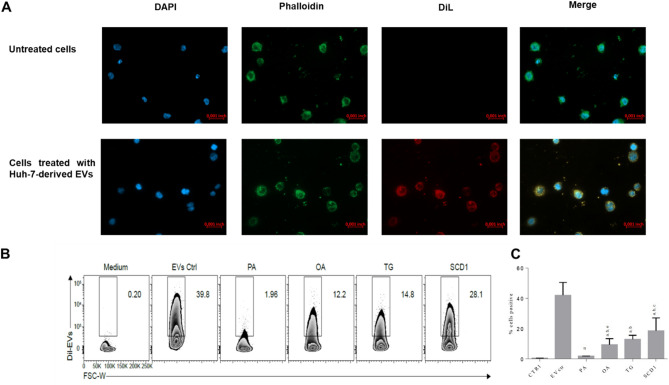
Uptake of EVs released by Huh-7 cells treated with fatty acids (PA or OA), Tg or SCD1i by THP-1 monocytes. (**A**) In order to demonstrate the internalization of EVs by THP1 we shown only the uptake of EVs derived from control cells. THP-1 cells were exposed to labeled Huh-7-EVs and, after 6 h, cells were fixed, actin filaments stained with FITC-labelled phalloidin and nuclei were counterstained with DAPI. The images are representative of one out of three separate experiments. 0.001 inch = 0.0254 mm; magnification 40 × . (**B**) Representative flow cytometry histogram plot showing the percentage of DiL-labeled EVs positive THP-1 cells. (**C**) The percentage of THP-1 cells taking up EVs released by control and treated Huh-7 cells was evaluated by flow cytometry after incubation with DiL-labeled EVs. Data represent the mean ± SD of three experiments after subtracting the background; *p* < 0.001; Two-way ANOVA, Sidak's multiple comparisons test performed with GraphPad software (a vs CTRL, b vs PA, c vs OA, d vs Tg, e vs SCD1i and π vs all).

The internalization of the EVs released by Huh-7 cells under membrane lipid saturation and/or ER stress conditions was evaluated in THP-1 monocytes. Intracellular localization of DiL-labeled EVs in THP-1 monocytes was examined by microscopy and flow cytometry (Fig. 8). After 6 h incubation, the DiL-fluorescence signal was detected in the cytoplasm indicating that CTRL-EVs were internalized in THP-1 monocytes (Fig. 8A). The evaluation of EV uptake efficiency by flow cytometry demonstrated that CTRL-EVs were taken up by THP-1 monocytes with a ~ 40% efficiency. The internalization efficiency of EVs released by Huh-7-treated cells was remarkably reduced compared to CTRL-EVs (Fig. 8B,C). Interestingly, the internalization of PA-EVs by THP-1 monocytes was almost completely abrogated (Fig. 8B,C).

## Discussion

The present study shows that Huh-7 cells translate changes in their membrane phospholipids to their released EVs. PA and SCD1i treatments induced ER stress and modified not only the phospholipid asset of cell membranes but also phospholipid composition of released EVs. The ER stressor Tg induced changes in EV lipid profiles similar to those observed in SCD1i-EVs. Interestingly, treatment of Huh-7 cells with the monounsaturated fatty acid OA did not induce relevant changes in lipid profiles of released EVs. These results suggest that the level of cell membrane saturation and/or ER stress are crucial to determine which lipids are discarded via EVs. The altered abundance in specific lipids observed in EVs released under hepatic lipotoxic conditions may not only reflect the state of lipid metabolism of parental cells but also give us insights into functional lipids that could be transported between cells via EVs. For example, EVs released by Huh-7 cells treated with PA are enriched in glycerophospholipids containing saturated and short fatty acids, SM and Cer, as compared to CTRL-EVs. Considering that PA-treated cells release higher amount of EVs, our findings indicate that PA-treated cells release via EVs a greater amount of specific lipids, i.e. sphingolipids and glycerophospholipids enriched in saturated and short fatty acids, that could both alleviate ER stress and affect lipid signaling in target cells. The comparison between lipids in EVs and parental cells also shows that fully saturated phospholipids tend to accumulate more in EVs than in cells after lipotoxic treatment as proven by the high concentration of PC 30:0, PC 32:0 and PC 34:0 in PA-EVs and SCD1i-EVs. The increased release of EVs carrying saturated phospholipids under lipotoxic conditions could represent a route used by hepatic cells to decrease the overload of saturated fatty acids, a condition that induces ER stress and an impairment of lipid metabolism. Interestingly, the other two treatments inducing ER stress, SCD1i and Tg, modified the phospholipid asset of EVs but did not modify the amount of released EVs. Noteworthy, all treatments decreased EV internalization efficiency by THP-1 monocytes, indicating that, under lipotoxic and/or ER stress conditions, Huh-7 cells released EVs with an altered lipid composition and different targeting properties.

Focusing the attention on effects exerted by the individual treatment on phospholipid composition of cells, we observed that PA induced rearrangements in PC and PE species, with an increase of molecular species containing saturated and short fatty acids. PA supplementation increased the level of Cer 16:0 and 18:0 consistently with known PA role as a precursor of de novo synthesis of Cer^28^. Therefore, this finding suggests that Huh-7 cells could employ PA for de novo synthesis of phospholipids, in agreement with previous evidence in primary hepatocytes and in the rat hepatoma H4IIEC3 cells^29^. The comparison with the other treatments, which also induced ER stress, showed that SCD1i increased the level of PC molecular species containing saturated and short acyl chains. The third ER stressor Tg, increased the level of PC containing short fatty acids. These observations hint that the PA, Tg or SCD1i induced ER stress could inhibit the fatty acid elongation occurring by a family of ER-resident enzymes. Previous evidence in MDBK cells demonstrate that Tg treatment inhibits the expression of fatty acid elongase 6^30^, an enzyme that specifically catalyzes the elongation of C12-16 saturated and monounsaturated fatty acids. OA supplementation also induced several rearrangements in phospholipid acyl chains, but the only relevant change was the increased levels of PEs containing short fatty acids. The global lipid analysis by PLS-DA (Fig. 7A) showed that all treatments induced specific changes in cellular phospholipids that allowed their discrimination in five separate clusters. A cellular overload of saturated fatty acids, similar to that induced by PA supplementation, could be induced by inhibition of SCD1, a SCD isoform that is highly expressed in lipogenic tissues, i.e. liver and adipose tissue^31^. Here, we demonstrated that the SCD1 inhibition in Huh-7 cells increased the level of Cer and of glycerophospholipids containing saturated and short fatty acids. The observed changes in saturation/elongation of the most abundant membrane phospholipids correlated well with previous studies showing that SCD1 inhibition significantly modified membrane phospholipid composition by regulating PC and PE synthesis^32^.

Despite the evidence that phospholipid asset changes in EVs released under lipotoxic conditions modify significantly their biological properties, to the best of our knowledge, no studies have investigated the complete lipid profile of EVs released by hepatocytes under increased membrane saturation and/or ER stress conditions. Here, we observed that PA supplementation and inhibition of SCD1 determined higher levels of saturated PC in the released EVs, compared to CTRL-EVs. SCD1i-EVs displayed also increased levels of PC species containing short fatty acids, while PA-EVs higher levels of PE containing short fatty acids. PA-EVs, SCD1i-EVs and Tg-EVs were enriched in SMs, compared to CTRL-EVs and OA-EVs. Further, the PA-induced increase of Cer(s) in cells and released EVs is in agreement with the higher levels of hepatic and circulating Cer previously observed in NAFLD^33,34^ and the PA-triggered release of Cer-enriched EVs from hepatocytes^19,20^. The global lipid profile analysis by PLS-DA (Fig. 7B) and Random Forest (Fig. 2S) indicate that CTRL-EVs and OA-EVs had a similar lipid asset, whereas PA-EVs showed a characteristic lipid composition that accounted for its clusterization in a separate group. The PA- EV enrichment in specific lipids, such as Cer 16:0 and 18:0, explains their clusterization in a separate group with respect to EVs released under ER stress condition induced by Tg or SCD1 inhibition.

Lyso-glycerophospholipids (lyso-PL) represent an interesting lipid class that was altered in the EVs released by Huh-7 cells under increased membrane saturation conditions. Lyso-PL are not only minor species produced during the synthesis and remodeling of membrane phospholipids but they are extracellular mediators involved in various biological processes by their action on cognate G protein-coupled receptors^35^. The presence of lyso-PL in EVs has been demonstrated in several studies^4,11,36^; lyso-PL present on the membrane of circulating EVs could activate lyso-PL receptors on the surface of target cells, mediating biological responses^37^. In this study, we demonstrated that PA and SCD1i treatments increased levels of saturated species of LPC, LPE and LPS in both cells and EVs. Considering that the treatment of hepatic cells with LPC induces the release of CXCL10-containing EVs, which are chemotactic for macrophages^17^, we could hypothesize that PA-EVs and SCD1i-EVs, presenting increased levels of LPC on their membrane could amplify the inflammatory responses by triggering the release of EVs in neighboring cells.

The EV internalization assay demonstrated that EVs released by PA, OA, SCD1i and Tg-treated cells were all internalized with lower efficiency, compared to CTRL-EVs. The uptake of EVs has been evaluated using THP-1 monocytes, a cell line widely used to study monocyte and macrophage functions/responses^38^, as target cells. Interestingly, the internalization of PA-EVs was almost completely abrogated. These results suggest that the internalization efficiency of EVs may be affected by their membrane lipid composition, which, in turn, could modify the asset of proteins present on the EV surface. In fact, EV targeting and uptake by recipient cells involve several proteins^39^ and specific lipid classes^40^ present on the surface of EVs. The concept that changes in EV lipid asset could have an impact on EV internalization is supported by a study demonstrating that the fusion efficiency of tumor Ex is strictly correlated to membrane rigidity and to the sphingolipid amount on EV membranes^41^. To date, the only study investigating the internalization of EVs released by PA-treated hepatocytes demonstrate a crucial role played by Vanin-1 present on EV surface, without reporting the internalization efficiency of PA-EVs in comparison with CTRL-EVs^42^. Nonetheless, it has been theorized that lipotoxic EVs might exert their biological effects without being internalized. Consistently with such a hypothesis, previous results demonstrate that EVs released by PA-treated hepatocytes carry on their surface molecules, such as S1P and TRAIL which activate macrophages acting on S1P or DR5 receptors, respectively^18,19^. Further studies are needed to investigate whether lipid composition influences directly or indirectly, by modifying proteins recruited on the EV membrane, EVs recognition and internalization by target cells. The combination of lipidomic and proteomic analysis will provide a complete picture of the molecular cargo of lipotoxic EVs and will improve the understanding of their biological functions. Moreover, useful information might derive from the evaluation of EV uptake in other cell types and, in particular, in cells involved in the pathogenesis of NAFLD. (i.e. HSC).

In conclusion, the present study reported that all treatments inducing ER stress modify lipid composition of released EVs. ER stress triggered by SCD1i and Tg induced similar changes in lipidomes of released EVs, whereas specific alterations in lipid composition distinctly characterized PA-EVs. Noteworthy, only PA increased EV release of several-fold. Moreover, the fact that PA- EVs were enriched in Cer 16:0 and 18:0 suggests that the higher PA-induced EV release could be related to an enhanced de novo synthesis of Cer from supplemented fatty acid. In recent years, an increasing number of papers focused only on the lipid composition of cells to identify the lipids and mechanisms that cause lipotoxicity^43,44^. The present study pointed out the paramount importance of EV lipidome analysis in driving the understanding of the underlying processes and mechanisms of lipotoxicity.

## Materials and methods

### Materials

All culture reagents were from Thermo Fisher Scientific. All HPLC solvents were MS grade from Wako. Phospholipid standards were purchased from Avanti Polar Lipids. All other reagents were from Sigma-Aldrich.

### Cell cultures and treatments

Huh-7 cells were grown in Dulbecco’s modified Eagle’s medium (DMEM) containing 10% (v/v) heat-inactivated fetal bovine serum (FBS), 2 mM glutamine, 100 U/ml penicillin, 100 U/ml streptomycin, in a humidified atmosphere containing 5% CO_2_ at 37 °C. For experimental purposes, cells (2–3 × 10^6^) were seeded in 100 mm culture dishes and, after 24 h, the culture medium was carefully removed and cells were washed twice with phosphate-buffered saline (PBS). Then, cells were treated for 16 h with 400 µM fatty acids (PA or OA), or Tg (2.5 nM), or SCD1i (5 µM) or vehicle (0.2% ethanol + 0.01% DMSO). Palmitic acid (P5585, Sigma-Aldrich) and oleic acid (O1008, Sigma-Aldrich) were dissolved in ethanol at a stock concentration of 148 mM; final concentration of ethanol was 0.2% in the medium. Tg (T9033, Sigma-Aldrich) and SCD1i (sc-205109A, Santa Cruz Biotechnology) were dissolved in DMSO at a stock solution of 2 mM and 25 mM, respectively; final concentration of DMSO was ≤ 0.01% in the medium. All treatments were carried out in serum-free medium containing 0.1% free fatty acids-Bovine Serum Albumin. Media were collected to isolate EVs by a differential centrifugation^26,45^ (see below). Cells were recovered and counted by Countess TC20 automated cell counter (Bio-Rad). Cell viability was assessed by Trypan Blue stain exclusion.

### Total RNA isolation and quantitative real-time PCR

Total RNA was extracted from cells using Isogen II (Nippon-gene) and reverse-transcribed using the High-Capacity cDNA Reverse-Transcriptase Kit (Applied Biosystems). Quantitative real-time PCR was carried out using the TaqMan Gene Expression System (Applied Biosystems) on SYBR Green PCR Master Mix (TaKaRa) and LightCycler 480 (Roche Diagnostics) or KAPA SYBR FAST qPCR Master Mix (NIPPON Genetics, Japan) and LightCycler96 (NIPPON Genetics, Japan). The probe-primer sets and the sequences of the oligonucleotides are as follows. C/EBP homologous protein (CHOP) forward (AGCTGGAAGCCTGGTATGAG) and reverse (GTGCGTGTGACCTCTGTTGG), DnaJ homolog, subfamily B, member 9 (DNAJB9) forward (ATCAGAGCGCCAAATCAAGAAGG) and reverse (TTCAGCATCCGGGCTCTTATTTTTG), growth arrest and DNA damage inducible protein 34 (GADD34) forward (GATGCCCTGGAGGAAGTGCT) and reverse (AGCAGGCACAACACCACGTT), glucose-regulated protein 78 (GPR78) forward (ACCGCTGAGGCTTATTTGG) and reverse (GCGTCTTTGGTTGCTTGG), glyceraldehyde-3-phosphate dehydrogenase (GAPDH) forward (GCCAAGGTCATCCATGACA ACT) and reverse (GAGGGGCCATCCACAGTCTT).

### Measurement of Xbox-binding Protein 1 (XBP1) mRNA Splicing

Total RNA was extracted from Huh-7 cells as described above. 1 µg of total RNA was reverse-transcribed with PrimeScript II 1st Strand cDNA synthesis Kit (TaKaRa Bio). PCR fragments representing the unspliced and spliced forms of XBP1 were amplified with Ex Taq polymerase (TaKaRa Bio). The following PCR conditions were used: 94 ˚C for 2 min → 30 cycle thermal cycle reaction of 94 ˚C for 30 s, 54 ˚C for 30 s, and 72 ˚C for 60 s → 72 ˚C for 7 min. Primer sequences used to amplify Human XBP1 were AAACAGAGTAGCAGCTCAGACTGC and TCCTTCTGGGTAGACCTCTGGGAG. The concentration of spliced and unspliced XBP1 amplified PCR products were quantified using MultiNA electrophoresis apparatus (Shimadzu) with DNA-1000 kit and ΦX174 DNA/HaeIII Markers (Shimazu). Electropherogram was integrated to obtain band intensity of each DNA fragment. XBP1 mRNA splicing % values were calculated as follows. Molar ratio of *sXBP1*/*allXBP1* = (*sXBP1* (nM) + ½ × *hXBP1* (nM) ) / (*sXBP1* (nM) + *uXBP1* (nM) + *hXBP1* (nM)). *hXBP1*: hybrid-*XBP1*, *sXBP1*: spliced-*XBP1*, *uXBP1*: unspliced-*XBP1*.

### EV isolation

Huh-7 cells were treated for 16 h with fatty acids, or Tg, or SCD1i or vehicle (CTRL) (2 dishes for each treatment), as described above. Then, media were recovered and subjected to low-speed centrifugations to remove cells, cell debris and large EVs (300 g for 10 min; 2,000 g for 10 min; 10,000 g for 30 min). Supernatants were further ultracentrifuged at 100,000 g for 70 min to pellet small EVs, which was washed in PBS and centrifuged again at 100,000 g for 70 min^26,45^. The final EV pellets were re-suspended in small volume of PBS (150–200 ml) and used for further analyses. Protein concentrations were determined by the Pierce BCA Protein assay kit (ThermoFisher, Carlsbad, CA, USA), using Bovine Serum Albumin as standard.

### Scanning electron microscopy

For scanning electron microscopy (SEM) examination were carried out according to previous study^4^. EVs were fixed in 2.5% glutaraldehyde for 15 min at room temperature, washed twice with large volume of water using Vivaspin concentration devices (300,000 Da cut-off), then sedimented on glass coverslips and allowed to dry at room temperature. SEM images were obtained using a field emission LEO 1525 electron scanning microscope (Zeiss; Thornwood, NY, USA) equipped with a Gemini column, after Cr metallization using a high-resolution sputter Q150T ES-Quorum apparatus (24 s. sputter at a current of 20 mA). Chromium thickness was ~ 10 nm.

### Nanoparticle tracking analysis

Nanoparticle tracking analysis (NTA) has been performed in order to assess the size distribution and the number of released EVs after the different treatments. EVs have been isolated from cell culture medium by differential centrifugation and the final pellet resuspended in the amount of PBS (filtered through a 0.02 µm Anotop 25 filter) needed to obtain a concentration within the recommended range (2 × 10^8^–1 × 10^9^ particles per ml). Samples have been vortexed for 1 min and then loaded into a NS500 instrument (Malvern Instruments Ltd, Worcestershire, UK). For each sample, 5 videos of 60 s were acquired and then processed using the NTA2.3 software. In this way, particles moving under Brownian motion are tracked and their hydrodynamic diameter is calculated using the Stokes–Einstein equation.

### Western blotting

Cells were recovered by centrifugation and pellets were resuspended in lysis buffer (62 mM Tris–HCl pH 6.8, 11% glycerol, 1% SDS containing phosphatase and protease inhibitors) and sonicated to prepare cell lysates.

Aliquots of cell lysates (15 μg proteins) or EVs (2 μg proteins) were mixed with sample buffer 5X (1 M Tris–HCl pH 6.8, 5% (w/v) SDS, 6% (v/v) glycerol, 0.01% (w/v) Bromophenol blue). Samples were electrophoresed on 12% acrylamide gel and electrotransferred to PVDF membrane. After blocking, membranes were incubated overnight with the following primary antibodies: anti-Alix (Santa Cruz, USA), anti-CD63 (Cymbus Biotechnology, UK), anti-β-actin (Sigma-Aldrich, USA), (Santa Cruz, USA), anti-calnexin (Stressgen, USA), anti-flotillin (BD Biosciences, Franklin Lakes, USA), anti-Apo B (EMD Millipore Corp, USA). HRP-linked secondary antibodies (GE Biosciences, Piscataway, USA) were probed according to manufacturer’s instructions. Immunoblots were detected by chemiluminescence using ECL system (GE Biosciences).

### Lipid analysis by liquid chromatography-tandem mass spectrometry (LC–MS/MS)

For lipid analysis, aliquot of cells (400 µg proteins) and EVs (20 μg of proteins) were used for lipid extraction according to Bligh-Dyer method^46^ with minor modification. Extracts were dried up and resuspended in IPA/methanol prior to be submitted for LC–MS/MS analysis.

For the detection of lipids, LC–MS/MS–based lipidomics analyses were performed on a Shimadzu Nexera UPLC system (Shimadzu) coupled with a QTRAP 4500 hybrid triple quadrupole linear ion trap mass spectrometer (AB SCIEX) as previously described^47^. Briefly, lipids extracted from cells or EVs were injected by an autosampler; typically, 10 μl (3 nmol phosphorous equivalent) of the sample was applied. Chromatographic separation was performed on a SeQuant ZIC-HILIC PEEK coated column (250 mm × 2.1 mm, 1.8 µm; Millipore) maintained at 50 ºC using mobile phase A (water/acetonitrile (95/5, v/v) containing 10 mM ammonium acetate) and mobile phase B (water/acetonitrile (50/50, v/v) containing 20 mM ammonium acetate) in a gradient program (0–22 min: 0% B → 40% B; 22–25 min: 40% B → 40% B; 25–30 min: 0% B) with a flow rate of 0.5 ml/min. The instrument parameters for negative ion mode were as follows: curtain gas, 10 psi; collision gas, 7 arb. unit; ionspray voltage, -4500 V; temperature, 700 ºC; ion source gas 1, 30 psi; ion source gas 2, 70 psi. The instrument parameters for positive ion mode were as follows: curtain gas, 10 psi; collision gas, 7 arb. unit; ionspray voltage, 4500 V; temperature, 700 ºC; ion source gas 1, 30 psi; ion source gas 2, 50 psi. MRM analysis of each lipid species was performed with the following transitions (Q1 and Q3): ([M + H]^+^, 184) for PC and SM, ([M + H]^+^, neutral loss of 141) for PE, ([M + H]^+^, 264) for ceramides, ([M − H]^−^, 241) for PI, ([M − H]^−^, neutral loss of 87) for PS, ([M − H]^−^, 153) for PG and PtdA.

### Evaluation of EV internalization

For EVs labeling, particles have been normalized according to their number/µg of protein, and then 1 × 10^8^ vesicles were stained for 30 min with 50 µM 1,1′-Dioctadecyl-3,3,3′,3′-Tetramethylindocarbocyanine Perchlorate (DiL; Thermofisher Carlsbad, CA, USA). The excess of dye was removed by washing EVs with PBS by ultracentrifugation at 100,000 × g for 70 min^48^. The pellet was suspended in fresh PBS. For fluorescence microscopy, THP-1 cells (ATCC TIB-202, 1 × 10^4^cells) were exposed to labelled EVs (~ 1 × 10^8^ nanovesicles) for 6 h. Then, cells were fixed with 4% paraformaldehyde for 20 min and F-actin was stained with fluorescein isothiocyanate (FITC)-labelled phalloidin (1:250) for 30 min and cell nuclei were counterstained with 4′,6′-diamidino-2phenylindole (DAPI). Cells were then rinsed in PBS, mounted and analysed with a Zeiss Axio Observer Z1 equipped with Apotome and digital Camera Axiocam MRm (Zeiss, Oberkochen, Germany). The images are representative of one out of three separate experiments (Magnification 40 ×).

The uptake percentage of DiL-labeled EVs positive cells were determined by flow cytometry. After 6 h incubation with labelled EVs, THP-1 cells were washed with 10 ml of FACS buffer and recovered by centrifugation. Then, cell pellets were resuspended in 500 μl of FACS buffer containing with 4% paraformaldehyde. Samples were run on LSRFortessa (BD Biosciences) flow cytometer and analyzed using FlowJo analysis software (Tree Star). Each experimental point was performed in triplicates.

### Statistical analysis

Lipid quantitative data are presented as mean ± SD of five independent experiments. Student’s test was applied to determine significant differences between two groups (*p* < 0.05) whereas ANOVA, followed by Tukey–Kramer post hoc test was applied to evaluate statistical differences between multiple treatment groups and controls (R statistical software, v3.6.1). Statistical significance was accepted when *p* < 0.05. The Partial Least Squares-Discriminant Analysis (PLS-DA), a multivariate regression method, was applied on the cleaned and log transformed dataset to extract via a linear combination of metabolites the information that can predict the categorical outcome (MetaboAnalystR package implemented in R statistical software v3.6.1^49^). Hierarchical cluster analysis, using Euclidean metric for distance measurement and ward.D clustering algorithm for hierarchical clustering, was also conducted by the MetaboAnalystR.

Random Forest analysis, a machine learning method to identify variables which best classify the data into different groups, was performed using the Random Forest package implemented in MetaboAnalystR.

## Supplementary Information

Supplementary Information 1.

Supplementary Information 2.
