# Supplementary material for: Lipotoxic stress alters the membrane lipid profile of extracellular vesicles released by Huh-7 hepatocarcinoma cells
Source: Sci Rep. 2021 Feb 25;11:4613. doi: 10.1038/s41598-021-84268-9 (PMC7907093; doi:10.1038/s41598-021-84268-9)
Supplement: Supplementary file 1 — Supplementary Information 1. [file 41598_2021_84268_MOESM1_ESM.docx]

**Original Western Blotting**

**Lipotoxic stress alters the membrane lipid profile of Extracellular Vesicles released by Huh-7 hepatocarcinoma cells**

Buratta S^1*#^, Shimanaka Y^2*^, Costanzi E^1^, Ni S^2^, Urbanelli L^1^, Kono N^2^, Morena F^1^, Sagini K^1,3^, Giovagnoli S^4^, Romani R^5^, Gargaro M^5^, Arai H^2, 6^, Emiliani C^1^

^1^Department of Chemistry, Biology and Biotechnology, University of Perugia, Perugia, Italy.

^2^Graduate School of Pharmaceutical Sciences, University of Tokyo, Tokyo, Japan.

^3^Department of Pharmaceutical Sciences, University of Perugia, Perugia, Italy.

^4^Department of Molecular Cell Biology, Institute for Cancer Research, Oslo University Hospital, Oslo, Norway

^5^Department of Experimental Medicine, University of Perugia, Perugia, Italy.

^6^AMED-CREST, Japan Agency for Medical Research and Development*.*

**^*^** These authors contributed equally to this work.

^#^ To whom the correspondence should be addressed: Sandra Buratta, sandra.buratta@unipg.it, Phone: + 39 075 585 7440.

**Original Image of Western Blotting**

**cells EVs**

**CD63 (Figure 1)**

**
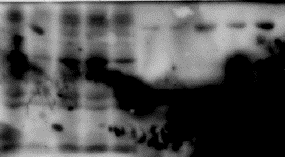
**

**Alix (Figure 1)**

**
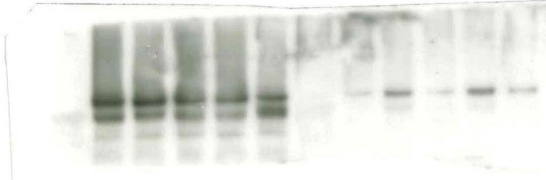
**

**Flot-1 (Figure 1)**

**
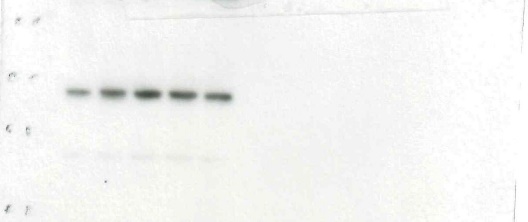

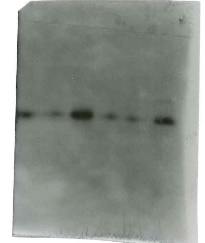
**

**Actin (Figure 1)**

**
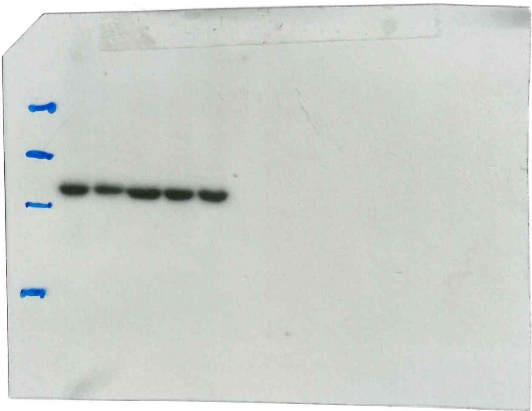
**

**Calnexin (Figure 1)**

**
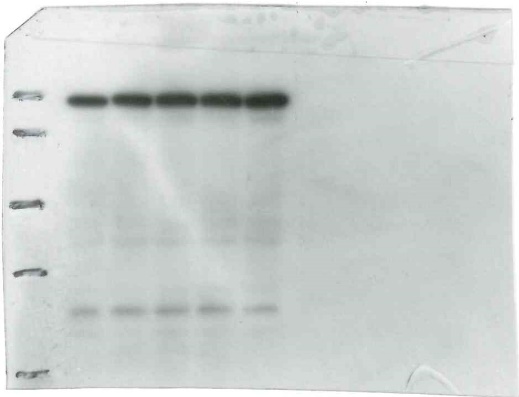
**

**ApoB 100 (Figure 2S)**

**
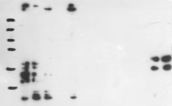
**

**CD63 (Figure 1S)**

**
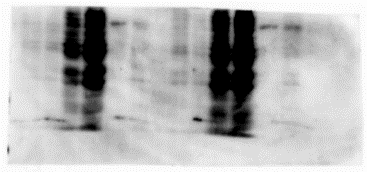
**

**Calnexin marker (Figure 1S)**

**
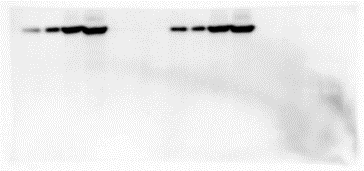
**
