# Supplementary material for: Lipotoxic stress alters the membrane lipid profile of extracellular vesicles released by Huh-7 hepatocarcinoma cells
Source: Sci Rep. 2021 Feb 25;11:4613. doi: 10.1038/s41598-021-84268-9 (PMC7907093; doi:10.1038/s41598-021-84268-9)
Supplement: Supplementary file 2 — Supplementary Information 2. [file 41598_2021_84268_MOESM2_ESM.docx]

**Supplementary Information**

**Lipotoxic stress alters the membrane lipid profile of Extracellular Vesicles released by Huh-7 hepatocarcinoma cells**

Buratta S^1*#^, Shimanaka Y^2*^, Costanzi E^1^, Ni S^2^, Urbanelli L^1^, Kono N^2^, Morena F^1^, Sagini K^1,3^, Giovagnoli S^4^, Romani R^5^, Gargaro M^5^, Arai H^2, 6^, Emiliani C^1^

^1^Department of Chemistry, Biology and Biotechnology, University of Perugia, Perugia, Italy.

^2^Graduate School of Pharmaceutical Sciences, University of Tokyo, Tokyo, Japan.

^3^Department of Pharmaceutical Sciences, University of Perugia, Perugia, Italy.

^4^Department of Molecular Cell Biology, Institute for Cancer Research, Oslo University Hospital, Oslo, Norway

^5^Department of Experimental Medicine, University of Perugia, Perugia, Italy.

^6^AMED-CREST, Japan Agency for Medical Research and Development*.*

**^*^** These authors contributed equally to this work.

^#^ To whom the correspondence should be addressed: Sandra Buratta


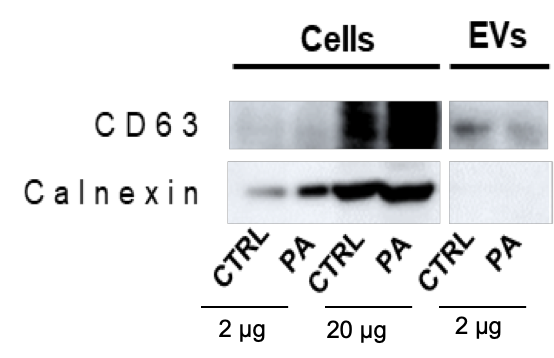


Figure 1S. Western blotting of marker (CD63) and non-marker (calnexin) of EVs. Cell lysates (2 or 20 μg) and EVs (2 μg) were separated by SDS-PAGE and immunoblotted with CD63 and calnexin antibodies as described in Materials and Methods.


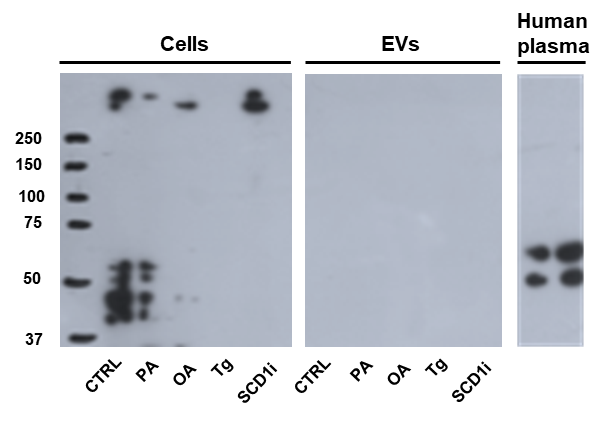


Figure 2S. Western blotting of apolipoprotein B. Cell lysates (20 μg) and EVs (2 μg) were separated by SDS-PGE (7% acrylamide) and immunoblotted with apolipoprotein B antibody. Human plasma was used as positive control.


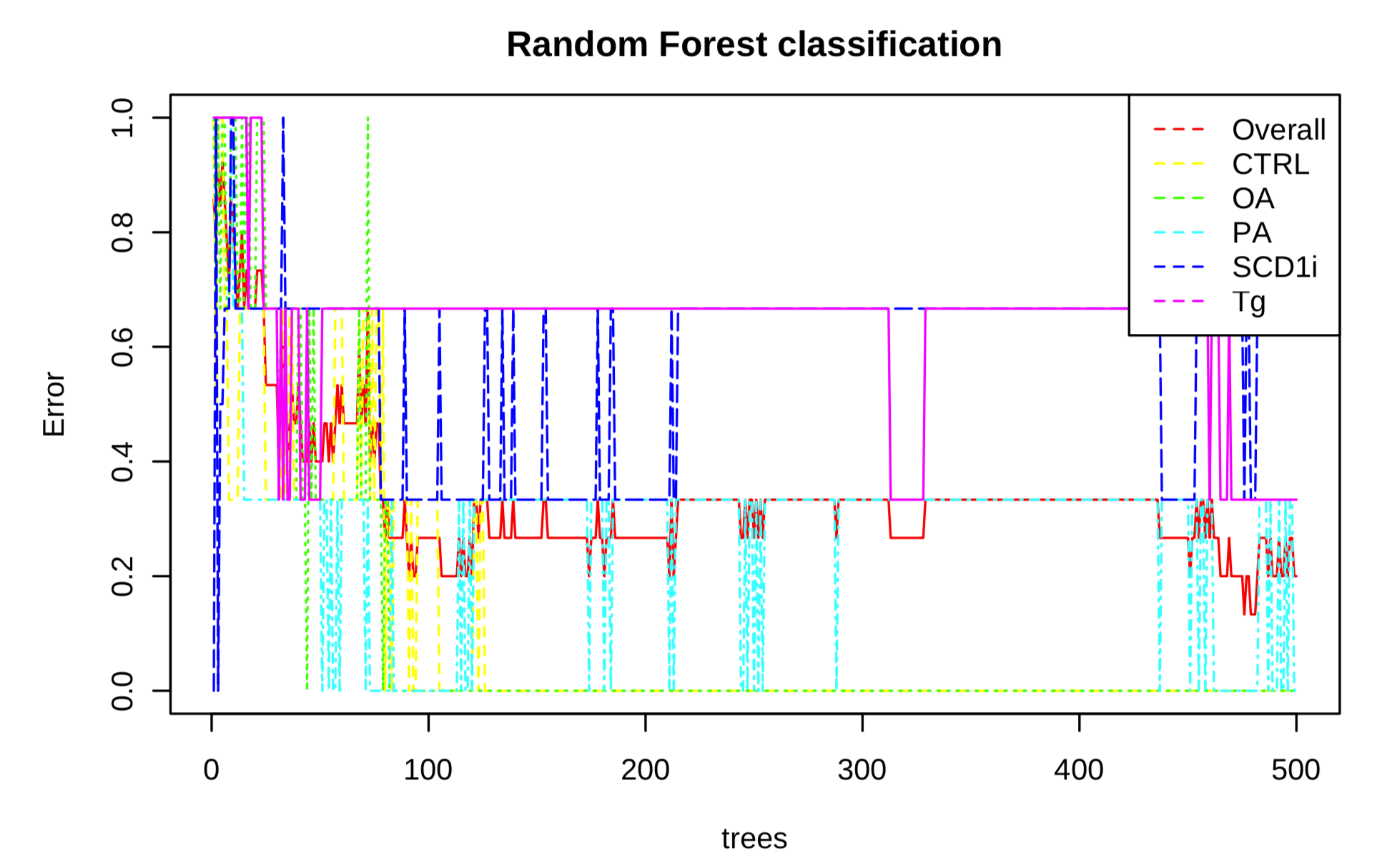


Figure 3S. Cumulative error rates by Random Forest classification in Huh-7 cells. The overall error rate is shown as the red line; the other lines represent the error rates for each class.

|  | CTRL | OA | PA | SCD1i | Tg | Class.error |
| --- | --- | --- | --- | --- | --- | --- |
| CTRL | 3.00 | 0.00 | 0.00 | 0.00 | 0.00 | 0.00 |
| OA | 0.00 | 3.00 | 0.00 | 0.00 | 0.00 | 0.00 |
| PA | 0.00 | 0.00 | 3.00 | 0.00 | 0.00 | 0.00 |
| SCD1i | 0.00 | 0.00 | 0.00 | 2.00 | 1.00 | 0.33 |
| Tg | 0.00 | 0.00 | 0.00 | 1.00 | 2.00 | 0.33 |

Table 1S. Random Forest Classification Performance. The out-of-bag error is 0.2


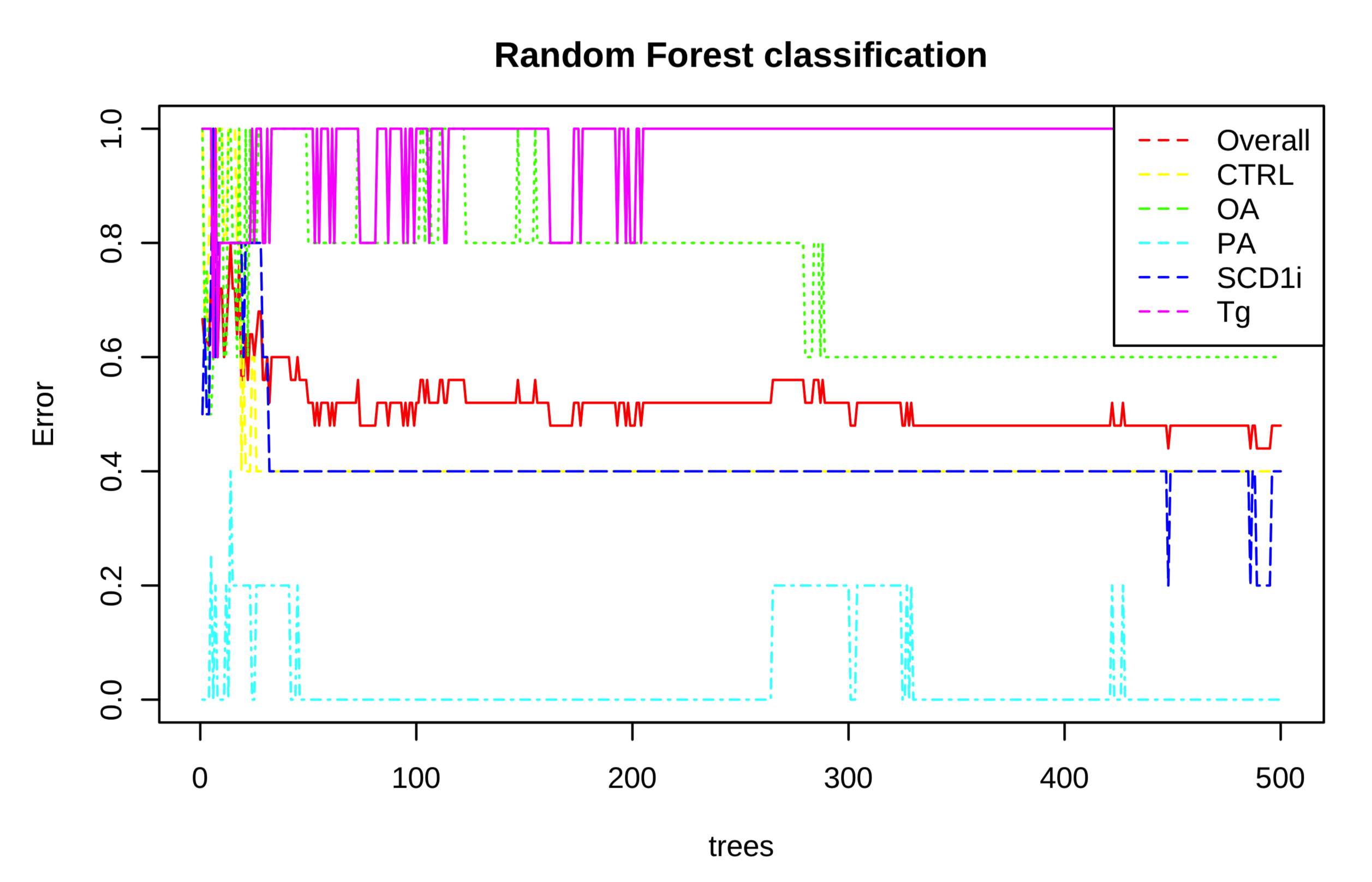


Figure 4S. Cumulative error rates by Random Forest classification in Huh-7-derived EVs. The overall error rate is shown as the red line; the other lines represent the error rates for each class.

|  | CTRL | OA | PA | SCD1i | Tg | Class.error |
| --- | --- | --- | --- | --- | --- | --- |
| CTRL | 3.00 | 2.00 | 0.00 | 0.00 | 0.00 | 0.40 |
| OA | 3.00 | 2.00 | 0.00 | 0.00 | 0.00 | 0.60 |
| PA | 0.00 | 0.00 | 5.00 | 0.00 | 0.00 | 0.00 |
| SCD1i | 0.00 | 0.00 | 1.00 | 3.00 | 1.00 | 0.40 |
| Tg | 2.00 | 0.00 | 0.00 | 2.00 | 1.00 | 0.80 |

Table 2S. Random Forest Classification Performance. The out-of-bag error is 0.42
